# Computational Investigation of Bisphosphate Inhibitors of 3-Deoxy-d-*manno*-octulosonate 8-phosphate Synthase

**DOI:** 10.3390/molecules24132370

**Published:** 2019-06-27

**Authors:** Jéssica de Oliveira Araújo, Alberto Monteiro dos Santos, Jerônimo Lameira, Cláudio Nahum Alves, Anderson Henrique Lima

**Affiliations:** Laboratório de Planejamento e Desenvolvimento de Fármacos, Universidade Federal do Pará, Belém 66075-110, Brasil

**Keywords:** KDO8P synthase, *Neisseria meningitidis*, bisphosphate inhibitors, molecular dynamics

## Abstract

The synthase, 3-deoxy-d-*manno*-octulosonate 8-phosphate (KDO8P), is a key enzyme for the lipopolysaccharide (LPS) biosynthesis of gram-negative bacteria and a potential target for developing new antimicrobial agents. In this study, computational molecular modeling methods were used to determine the complete structure of the KDO8P synthase from *Neisseria meningitidis* and to investigate the molecular mechanism of its inhibition by three bisphosphate inhibitors: BPH1, BPH2, and BPH3. Our results showed that BPH1 presented a protein–ligand complex with the highest affinity, which is in agreement with experimental data. Furthermore, molecular dynamics (MD) simulations showed that BPH1 is more active due to the many effective interactions, most of which are derived from its phosphoenolpyruvate moiety. Conversely, BPH2 exhibited few hydrogen interactions during the MD simulations with key residues located at the active sites of the KDO8P synthase. In addition, we hydroxylated BPH2 to create the hypothetical molecule named BPH3, to investigate the influence of the hydroxyl groups on the affinity of the bisphosphate inhibitors toward the KDO8P synthase. Overall, we discuss the main interactions between the KDO8P synthase and the bisphosphate inhibitors that are potential starting points for the design of new molecules with significant antibiotic activities.

## 1. Introduction

The 3-deoxy-d-*manno*-octulosonate 8-phosphate (KDO8P) synthase is an important enzyme in the lipopolysaccharide (LPS) biosynthesis of gram-negative bacteria [1]. It catalyzes the aldol-type condensation of d-arabinose 5-phosphate (A5P) and phosphoenolpyruvate (PEP), forming 3-deoxy-d-*manno*-octulosonate-8-phosphate (KDO8P) and inorganic phosphate (Pi) [2]. The compound, 3-deoxy-d-*manno*-octulosonate (KDO), is an essential component of the lipopolysaccharide layer found in the cell walls of all gram-negative bacteria [3,4]. The absence of KDO causes the disruption of LPS, leading to severe alteration of the growth and normal functions of gram-negative bacteria [1,5,6,7]. The KDO pathway is present only in gram-negative bacteria and plant species, and it is considered a potential target for the development of new anti-microbial drugs [8].

The KDO8P synthase belongs to the family of transferases and can be divided into two classes based on the presence or absence of a metal ion in its active site [9,10]. The KDO8P synthases from *Escherichia coli* and *Neisseria meningitidis* are metal independent enzymes [2,11,12,13], while those from *Aquifex aeolicus, Aquifex pyrophilus, Helicobacter pylori, Acidithiobacillus ferrooxidans*, and *Chlamydia psittaci* require a metal ion to perform their catalytic activities [9,14,15,16,17]. Despite the metal dependency, the active sites of these enzymes are practically identical; they differ only in the substitution of cysteine, the amino acid involved in the metal coordination, with asparagine in the metal-independent enzyme [10]. 

The catalytic mechanism of the KDO8P synthase leads to the formation of a double-phosphorylated intermediate, after which the products, KDO8P and Pi, are formed [18,19,20,21]. Thus, monophosphate and bisphosphate molecules have been studied to construct new potential inhibitors that mimic the tetrahedral intermediate of the reaction. These tetrahedral intermediate derivatives maintain the stereochemistry of the molecule and the charged phosphate groups derived from both PEP and A5P substrates [12,22]. 

**Scheme 1:**
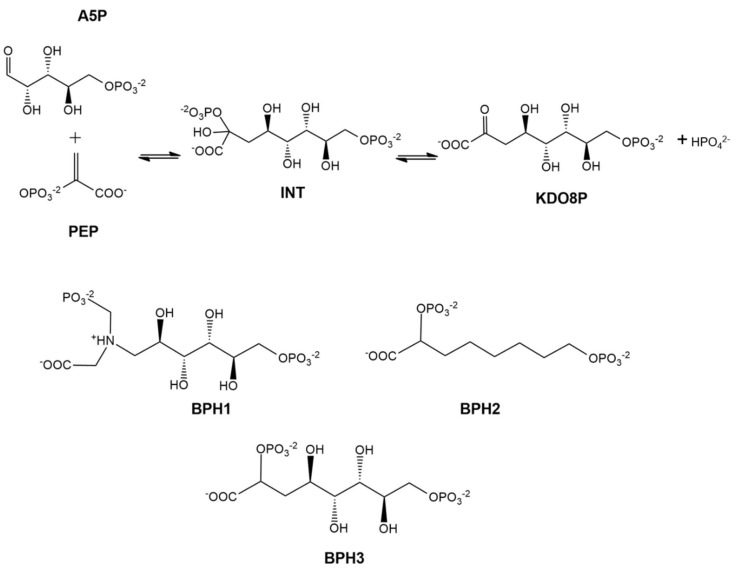
At the top, 2D structures that represent the reaction of d-arabinose 5-phosphate (A5P) and phosphoenolpyruvate (PEP) to form 3-deoxy-d-*manno*-octulosonate-8-phosphate (KDO8P) and inorganic phosphate (Pi). The doubly phosphorylated intermediate (INT) is used as a reference for the design of the analog inhibitors: BPH1, BPH2, and BPH3.

Several molecules have been explored as reaction intermediate mimics. Bisphosphate 1 (BPH1) [12] and bisphosphate 2 (BPH2) [22] are described in the literature as the best inhibitors of KDO8P synthase (Scheme 1). 

However, little is known about the mode of action of these inhibitors at a molecular level, particularly their interactions with the KDO8P synthase from *Neisseria meningitidis*. In this work, we performed molecular modeling of the inhibitors, BPH1 and BPH2, in complexes with the KDO8P synthase to comprehend the molecular biphosphate inhibitor interactions. In addition, we hydroxylated BPH2 to create the hypothetical molecule, named BPH3 (Scheme 1) to investigate the influence of the hydroxyl groups on the affinity of the bisphosphate inhibitors toward the KDO8P synthase. Molecular modeling methods can provide valuable information regarding the molecular basis for the bisphosphate-based inhibition of the KDO8P synthase from *Neisseria meningitidis* and to further contribute to the search for new antibacterial agents in gram-negative bacteria. 

## 2. Results and Discussion

### 2.1. Homology Modeling

**Figure 1:**
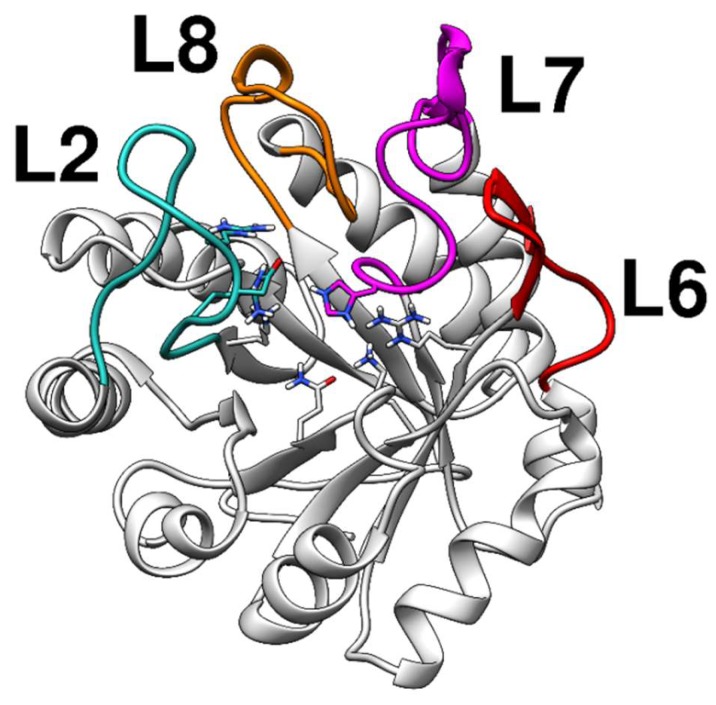
Structure of the modeled KDO8P synthase from *Neisseria meningitidis* evidencing the regions of the loops. The loops, L2, L7, and L8, control access to the active site.

The KDO8P synthase crystal structure from *Neisseria meningitidis* is currently available (PDB ID: 2QKF); however, the loops near the catalytic sites are absent. Since the metal-independent KDO8P synthase is generally difficult to analyze using X-ray diffraction techniques, we built, through homology modeling, a complete model of the KDO8P synthase based on the crystal structure of the KDO8P synthase from *Escherichia coli* (PDB ID: 1X6U). The main loops of the modeled KDO8P synthase are shown in Figure 1. 

After building the model, the structure was validated through different approaches. The stereochemical quality of the modeled protein determined by the Ramachandran plot analysis showed 91.82% of the residues in a favored region (Figure S1). The Anolea and QMEAN analyses presented great results as well. For more details, see Supporting Information (Figure S2). The overall and local model qualities were evaluated by ProSA-web to identify errors in the three-dimensional structures of the protein model. Thus, a Z-score of −7.6 points implies a good model since the quality is evaluated using solved protein structures as references (Figure S3). Finally, ERRAT was used to analyze the statistics of the non-bonded interactions between different atom types based on the characteristics of the atomic interactions [23]. The overall quality factor was determined as 94.574, which is very satisfactory (Figure S4).

Therefore, the modeled enzyme appeared to be a good starting point to study the interactions through docking and molecular dynamics simulations. Notably, the metal dependence of the KDO8P synthase has been extensively studied; recently, an evolutionary hypothesis showed that the catalytic activity of the metal-dependent KDO8P synthase is more compromised by the truncation of L7 than the metal-independent enzyme is [24]. The metal ion facilitates the correct coordination of A5P in the catalytic site; thus, metal independent enzymes are more reliant on the extended L7 loop for accurate A5P binding. 

### 2.2. Docking Analysis

The enzyme, KDO8P synthase, catalyzes the condensation reaction between PEP and A5P to produce KDO8P. First, we demonstrated that the bound conformations of PEP, A5P, and KDO8P could be reproduced in silico by MVD algorithms [25]. The results of these re-docking simulations are presented in Supplementary Information (Figure S5) and they show very small deviation from the reference crystal structure (0.15, 0.39, and 0.34 Å for PEP, A5P, and KDO8P, respectively). We used re-docking experiments with known complexes (the substrates: A5P, PEP; and the product: KDO8P), which were of similar conformational complexities with the inhibitors. This is usually performed to evaluate the docking protocol being used, as mentioned by Olson’s group [26]. The objective of the procedure is to verify that the docking parameters specified in the input file for the docking method are reasonable and are able to recover the structure and interactions of a known complex.

**Figure 2:**
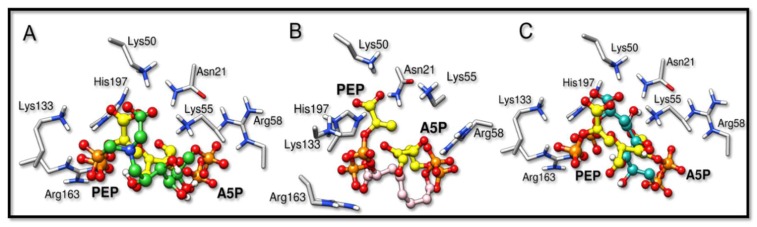
Overlapped structures of the bisphosphate inhibitors with PEP and A5P substrates (PDB ID: 1FXQ). The carbon atoms of the substrates are colored yellow. (**A**–**C**) are the superimpositions with BPH1, BPH2, and BPH3, respectively. It is noted that the phosphate groups derived from the substrates overlap the phosphate groups of the inhibitors.

The inhibitors studied in this work, BPH1, BPH2, and BPH3, share structural and functional similarities with both the A5P and PEP substrates [22,27]. Our results showed that these inhibitors bind similarly, as can be seen in the comparison with the structures of the homologous KDO8P synthase from *Aquifex aeolicus* (PDB ID: 1FXQ, Figure 2 and 1FWW, Figure S6) [28].

The binding modes of BPH1, BPH2, and BPH3 into the active sites of the KDO8P synthase are essentially the same at the PEP-moiety. The phosphate group of BPH1 interacts with Lys133 and Arg163, while the carboxylate group of BPH1 interacts with Lys50, Lys55, and His197. The residue, His197, which also interacts with the phosphate group of the PEP-moiety, is known to coordinate the divalent ion of the metal-dependent KDO8P synthase [28]. BPH2 and BPH3 interact similarly, differing only in their interactions with Lys50, where there is a shorter interaction with the carboxylate group of BPH3 than with that of BPH2.

### 2.3. Molecular Dynamics Simulations

**Figure 3:**
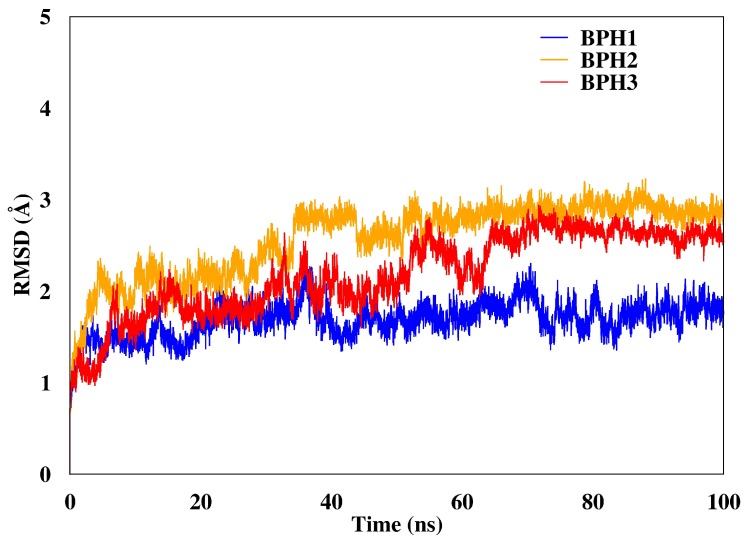
RMSD of the backbone atoms of the complexes after 100 ns of MD simulations. The RMSD represents the enzyme in the presence of the BPH1 (blue), BPH2 (yellow), and BPH3 (red) inhibitors.

We calculated the root mean square deviations (RMSD) of BPH1, BPH2, and BPH3 in the complexes with the KDO8P synthase along the 100 ns of MD simulation (Figure 3). The result highlighted the higher stability of the BPH1-KDO8P synthase complex compared to those of the BPH2 and BPH3 complexes. Moreover, BPH2 and BPH3 exhibited similar behaviors regarding their stabilities over the simulation. 

**Figure 4:**
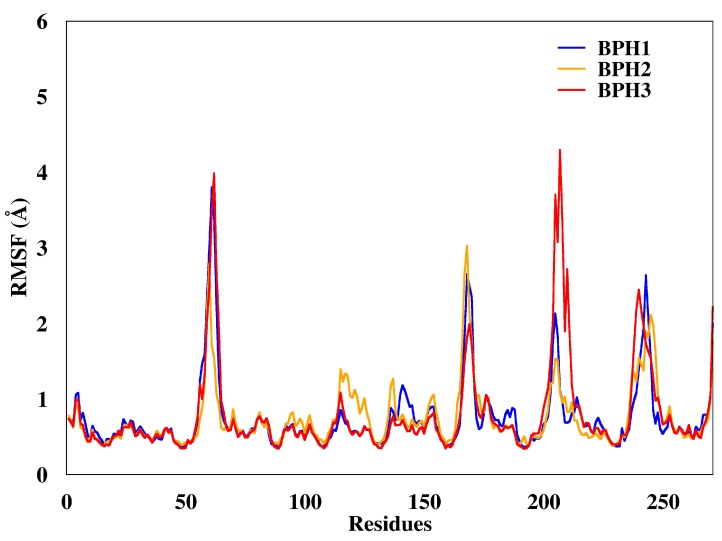
Root mean square deviations (RMSF) of the backbone atoms of the complexes from representative snapshots of MD simulations. Enzyme in the presence of the BPH1, BPH2, and BPH3 inhibitors are colored blue, yellow, and red, respectively.

Regarding the fluctuations observed for the three complexes, the BPH3-KDO8P synthase complex, particularly residues 200–218 (loop L8), showed slightly higher fluctuations (Figure 4) than others did.

It appears that the stability of the whole complex may be related to the distances of hydrogen bond interactions obtained from the molecular dynamic simulations. The inter-atomic distance obtained for the interaction between BPH1 and the residues at the catalytic site was shorter than the corresponding distances from the BPH2 (Table 1). Additional interactions have been observed for the BPH3-KDO8P synthase complex that differs from those of BPH1 and BPH2, but with similar contributions toward the stability.

**Figure 5:**
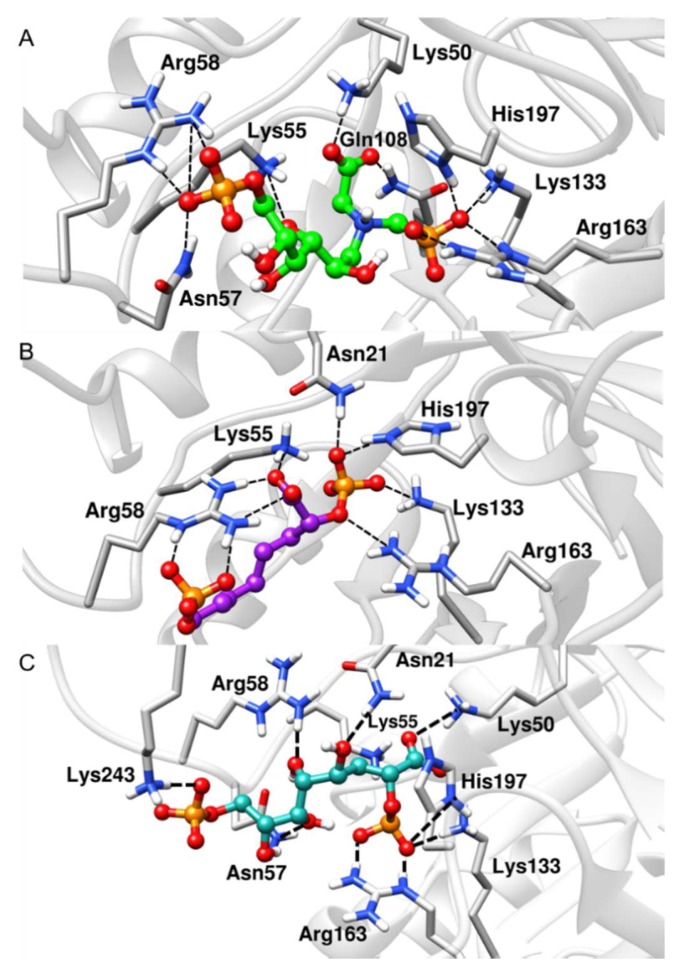
Representative structures along the MD simulations. (**A**) BPH1-KDO8P synthase, (**B**) BPH2-KDO8P synthase, and (**C**) BPH3-KDO8P synthase complexes. We can see important interactions of phosphate groups of the inhibitors with positively charged groups of KDO8P synthase.

The deviation data shown in Table 1 confirm the stability of the ligands along the simulations. Notably, the BPH3 inhibitor interacts with the hydroxyl groups that connect the two phosphate groups. Thus, the distance between the polar residues, Arg58 and Asn57, increases relative to the P1 atom when compared to those of the inhibitors, BPH1 and BPH2 (see Figure 5). 

**Figure 6:**
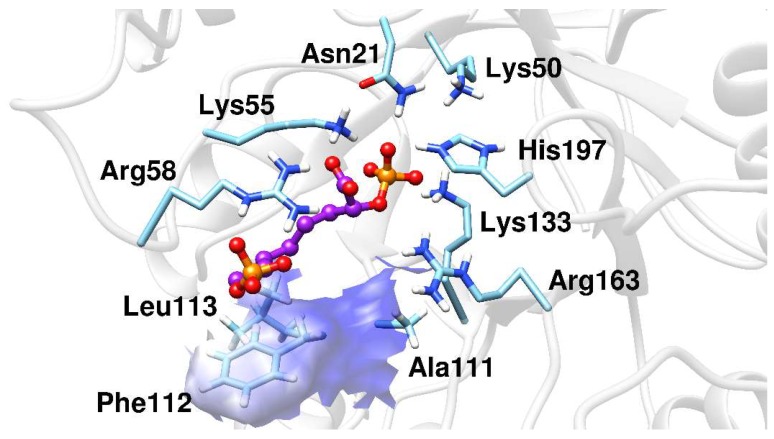
The BPH2 inhibitor having polar contacts at the active site of the KDO8P synthase.

The MD simulations results showed that BPH1 and BPH2 differ in their interactions on the A5P moiety. The portion of the BPH2 inhibitor that is located in the pocket of the A5P substrate starts to interact with the nonpolar residues, Pro110, Phe112, and Leu113, implying that BPH2 loses the interaction with some residues from loop L7, as can be seen in Figure 6. 

These interactions with nonpolar residues motivated the investigation on the effect of hydroxylating the chain that links both phosphate groups in the bisphosphate inhibitors. Our results showed that hydroxylated bisphosphate inhibitors, such as BPH3, may not only increase the number of interactions at the catalytic sites of the protein, but also contribute to self-conformational stabilization.

### 2.4. Binding Free-Energy Analysis

BPH1 is the best inhibitor of the series called “phosphate linkers” [29,30]. The binding free-energies (∆G_bind_) of BPH1, BPH2, and BPH3 in complexes with the enzyme, KDO8P synthase, have been computed during the last 1000 snapshots of the MD simulations. Table 2 shows the results for the global interaction energy for each complex.

As can be seen in Table 2, BPH1 showed higher affinity towards the KDO8P synthase than BHP2 did in the BPH2-KDO8P synthase complex. This result is corroborated by previously reported experimental results that showed a constant of inhibition of 0.37 μM for BPH1 [31] against 7.9 μM for BPH2 [22]. Moreover, the addition of hydroxyl groups to the BPH2 inhibitor increased the binding-receptor affinity, thereby increasing the proximity of the values to those obtained for the BPH1 inhibitor.

**Figure 7:**
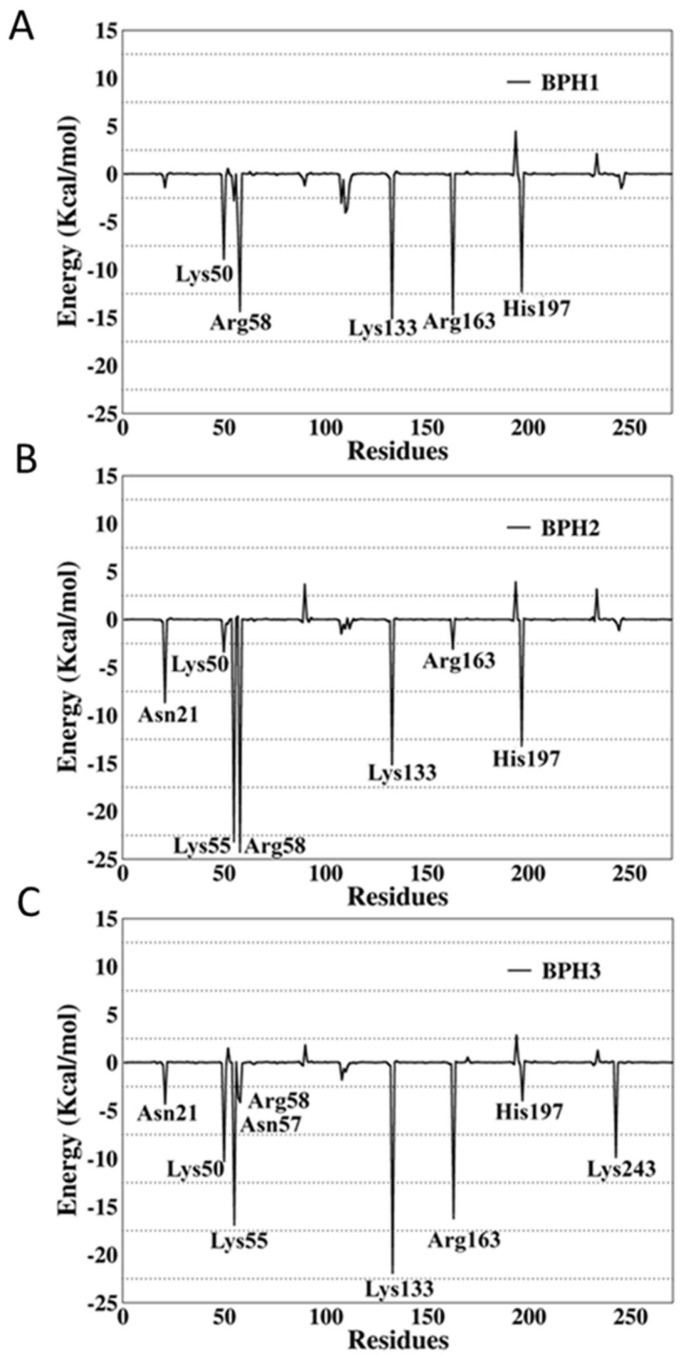
Per-residue decomposition analysis of (**A**) BPH1, (**B**) BPH2, and (**C**) BPH3 in complexes with the KDO8P synthase from *N. meningitidis*.

To verify the contribution of each residue to the global interactions with the inhibitors, the last 1000 snapshots obtained from the MD simulations trajectory were analyzed using the per-residue decomposition (MM-GBSA) approach. The results are illustrated in Figure 7, where negative values correspond to attractions, and the positive values to repulsions. 

The per-residue energy decomposition of the BPH1-KDO8P synthase complex indicated multiple interactions in the enzyme active sites. The key residues, Lys50, Arg58, Lys133, Arg163, and His197, contributed greatly with −8.9, −14.34, −15.65, −14.66, and −12.29 kcal/mol, respectively.

The interactions of BPH2 with Lys55 and Arg58 are more effective than those of BPH1 and BPH3 (Figure 7). The negatively charged phosphate group promotes an optimal interaction with the positively charged groups, particularly arginine and lysine residues at the active site.

Apparently, BPH1 undergoes many interactions, which result in a protein–ligand complex with the highest affinity, as can be seen in Table 2. Furthermore, the absence of polar contacts between the enzyme and the chain that links the phosphate groups of the inhibitor causes a repulsion observed mainly with the residue Asp90. The inclusion of hydroxyl groups in the hypothetical BPH3 molecule allows interactions with Asn21, Asn57, and Arg58. Besides, Lys243 appears to interact strongly with BPH3 (about −10 kcal·mol^−1^), an interaction, which is not observed in BPH1 and BPH2 complexes (See Figure 5 and Figure 7). 

**Figure 8:**
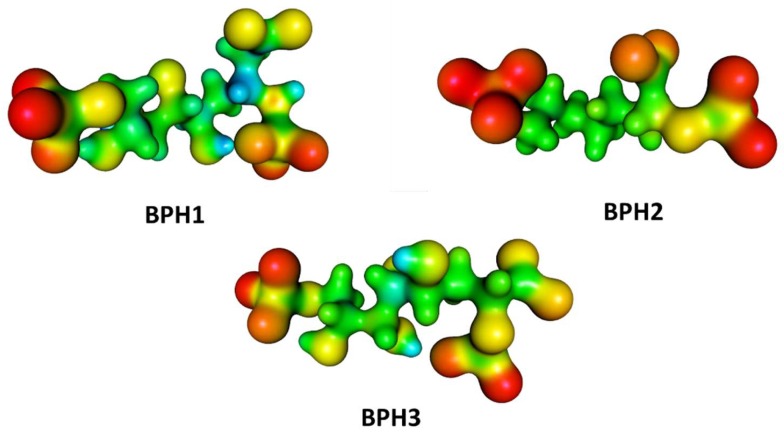
Molecular electrostatic potential (MEP) surfaces of biphosphate inhibitors BPH1, BPH2, and the hypothetical BPH3. Calculations were done at the M06-2X/6-31++G (d,p) level.

Furthermore, we evaluated the influence of the KDO8P synthase on the electrostatic potential surfaces of the inhibitors along the simulation, and determined the molecular electrostatic potential (MEP) surfaces for the inhibitors in a protein environment (Figure 8). The MEP surfaces were obtained from the M06-2X/6-31++G (d,p) level using single-point structures obtained from the MD simulations. These surfaces correspond to an isodensity value of 0.002 au. The most nucleophilic regions (negative electronic potential) are shown in red, while the most electrophilic regions (positive electrostatic potential) are shown in blue. Our results show that the regions of positive charge are predominantly on the hydroxyl groups of BPH1 and BPH3 (Figure 8). 

Conversely, the most negative charges are on the phosphate group of the A5P moiety. Notably, the biphosphate inhibitors resemble the oxocarbenium ion intermediates formed by the proposed intermediate derivatives that have long been targets of inhibitor design. This may provide a basis for the design of new inhibitors since analogs of the reaction intermediates can be used to inhibit the KDO8P synthase. In addition, note that BPH1 and BPH3 have a more positive charge around C2 in the protein than BPH2 does (see Figure 8). This positive potential around C2 is a consequence of both the electrostatic interaction between the enzyme and inhibitors and the electrostatic interaction with the substrates itself. Thus, as expected, the KDO8P synthase devotes its catalytic power to stabilizing its cationic intermediate. 

## 3. Materials and Methods 

### 3.1. Homology Modeling

The modeling of the systems was performed using the sequence of the enzyme, KDO8P synthase, from *Neisseria meningitidis* obtained from UNIPROT (ACC Q9JZ55) and submitted to the SWISS-MODEL server [32]. The metal-independent KDO8P synthase is generally difficult to discern by X-ray diffraction techniques, and therefore, some Protein Data Bank (PDB) structures do not exhibit important protein loop regions [10]. Thus, we built, by homology modeling, a complete model of the KDO8P synthase based on the crystal structure of the KDO8P synthase from *Escherichia coli* (PDB ID: 1X6U) [33]. Firstly, the most suitable template was explored for homology modeling. PDB ID 1X6U was preferred because it is 71.22% identical and it has the lowest e-value. After modeling the 3D structure, the quality and validation of the model were evaluated by several structure assessment methods including PROCHECK [34], where the overall stereochemical quality of the protein was assessed by the Ramachandran plot analysis [35], Anolea [36], QMEAN [37], z-score by ProSA-web [38], and ERRAT [23].

### 3.2. Molecular Docking Study

The molecular dockings of BPH1, BPH2, and BPH3 into the active site of the KDO8P synthase from *N. meningitidis* were simulated using a Molegro Virtual Docker (MVD). The MVD is a fast and flexible docking program that estimates the most probable binding conformation of a ligand to a macromolecule [25]. It includes MolDock, which is a heuristic search algorithm that combines differential evolution with a cavity prediction algorithm [25,39]. The docking scoring function of MolDock is an extension of the piecewise linear potential (PLP), including new hydrogen bonding and electrostatic terms. Further, a re-ranking scoring function is introduced to identify the most promising docking solution from the solutions obtained by the docking algorithm.

To develop the docking methodology, we first attempted to demonstrate that bound conformations could be reproduced in silico. For this purpose, A5P and PEP (substrates) and KDO8 (product) from the PDB ID 1FWW and 1X6U, respectively, were re-docked using the template docking feature implemented in the MVD program. The fitness evaluation of each re-docked pose was evaluated by considering the RMSDs values and docking scores. The selected re-docked pose was further evaluated for its interactions and energetic analysis to investigate the efficiency of the docking search algorithm and scoring function by comparing its values with those of the bound conformation.

In all the simulations (re-docking the known bound conformations and docking the bisphosphate inhibitors), a minimum of 10 runs was performed. The MolDock scoring function was set to a grid resolution of 0.30 Å and a maximum iteration of 3000 with a simplex evolution of size 100. Subsequently, the main interactions in the docking results were compared to those of the crystal structures for PEP and A5P (PDB ID: 1FXQ and 1FWW) [9]. The best pose of each inhibitor was selected for subsequent ligand-protein interaction energy analysis. The selection was performed based on smaller RMSD and lower binding energy values.

### 3.3. MD Simulations

Molecular dynamics simulations were performed to verify the stabilities of the complexes formed from the interaction between KDO8P synthase from *Neisseria meningitidis* and the ligands, BPH1, BPH2, and BPH3. The Amber 16 suite [40] combined with the Amber ff14SB force field was used to perform the MD simulations. Hydrogen atoms were added into the protein structure using the tLeap module, where the protonation states of charged protein residues were obtained from the H++ server (http://biophysics.cs.vt.edu/H++) [41]. The charges of the bisphosphate inhibitors were calculated on a Gaussian 09 program [42] using the Hartree–Fock method and 6-31G* basis set [43]. Thereafter, the system was solvated in a truncated octahedron TIP3P water box. The distance between the wall of the box and the closest atom of the solute was 12.0 Å. Counter ions (in this case, Cl^−^) were added to maintain the electroneutrality of the system. All the hydrogen atoms were minimized for 2000 steps of steepest descent, followed by 3000 steps of conjugate gradient. Next, the positions of the water molecules were relaxed using the same protocol. The whole system was energy-minimized for 5000 steps of steepest descent plus 5000 steps of conjugate gradients. Afterward, we started the thermalization of the system from 0 to 298 K running 100 ps molecular dynamics with position restraints at a constant volume. To equilibrate the system before production dynamics, we ran 500 ps molecular dynamics without positional restraints at a constant pressure. We performed 100 ns of MD simulation at a temperature of 298 K. The SHAKE algorithm [44] was used to maintain all the bonds at their equilibrium distances, which allowed the use of an integration time step of 2 fs. Finally, a cut-off of 12 Å was used for non-bonded interactions.

### 3.4. Free Energy Calculations

The molecular mechanical energy combined with the Poisson–Boltzmann or generalized Born and surface area continuum solvation (MM/PBSA and MM/GBSA) methods are popular approaches to estimating the free energy of the binding of small ligands to biological macromolecules [45,46]. In general, these methods estimate the binding free energy as follows:

(1) $$\Delta G_{bind}=G_{complex}-\left(G_{receptor}+G_{ligand}\right),$$

where $\Delta G_{bind}$ can be decomposed into the following three terms:

(2) $$\Delta G_{bind}=\Delta E_{MM}+\Delta G_{sol}-T\Delta S$$

The molecular mechanical energy ($\Delta E_{MM}$) represents the summation of the intramolecular energy; the solvation energy ($\Delta G_{sol}$) is composed of the polar ($\Delta G_{PB/GB}$) and non-polar contributions ($\Delta G_{SA}$); finally, the entropic contribution (TΔS) is associated with the conformational entropy loss when a free-state ligand binds to the corresponding unbound-state receptor.

Similar to MM/PBSA and MM/GBSA, the solvated interaction energy (SIE) [47] treats the protein–ligand system in atomistic detail and solvation effect simplicity. Each interaction and desolvation contribution are further made up of an electrostatic component and a nonpolar component:

(3) $$\Delta G_{bind}\approx E_{inter}+\Delta G_{desolv}=E_{inter}^{col}+\Delta G_{desolv}^{R}+E_{inter}^{vdW}+\Delta G_{desolv}^{np}$$

Thus, the electrostatic SIE component includes the Coulombic intermolecular interaction energy, $E_{inter}^{col}$, and the electrostatic desolvation free energy, $\Delta G_{desolv}^{R}$, due to the change in the reaction field energy upon binding. The nonpolar SIE component includes the van der Waals intermolecular interaction energy, $E_{inter}^{vdW}$, and the nonpolar desolvation free energy, $\Delta G_{desolv}^{np}$, that results from changes in the solute-solvent van der Waals interactions and changes during maintenance of the solute-size cavity in water.

Thereby, the free energies for the binding of the ligands were calculated from the MD trajectories using Amber16 for PB/GB-SA calculations; SIETRAJ was used to calculate the bind energies from the SIE method. For each complex, the binding free energy was estimated from snapshots taken from the trajectory at 1 ps intervals from the last 1000 snapshots. Additionally, we figured out the residues that most contribute to the calculated overall binding energy using a residue-by-residue decomposition protocol embedded in the GB solvent model based on the MMGBSA approach.

## 4. Conclusions

In this study, we employed docking, molecular dynamics, and binding free-energy calculations to provide insights into the molecular mechanism of metal-independent KDO8P synthase inhibition. In accordance with our results, BPH1 presented the protein–ligand (∆G_bind_) complex with the highest affinity, which is in agreement with experimental data that show it as the most efficient for inhibiting the metal-independent KDO8P synthase. Our results showed that this high affinity is due to the relatively high number of interactions with key residues at the active sites of the enzyme, particularly Lys50, Lys55, Arg58, Lys133, Arg163, and His197. Besides, hydroxylated carbons help the enzyme sustain polar contacts that are essential for maintaining the main interactions primarily with the A5P substrate. Therefore, this study provides a molecular interpretation of the main interactions that occur between the KDO8P synthase and bisphosphate inhibitors, thereby contributing to a better understanding of the inhibition processes involving this biological target and aiding the design of new inhibitors as candidates for antibiotics. We hope that the results reported herein may be useful for designing molecules with more interesting inhibitory activities on the basis of their three-dimensional structures.

## Figures and Tables

**Table 1:** Distances (in Å) for the phosphate groups from MD simulations. (P1) represents the P atom derived from the PEP moiety, while (P2) represents the P atom from the A5P moiety (see Figure S3 for details).

KDO8P Synthase (*N. Meningitidis*)							
	BPH1 (P1)	BPH2 (P1)	BPH3 (P1)		BPH1 (P2)	BPH2 (P2)	BPH3 (P2)
Arg58 (NH2)	2.8 (± 0.1)	2.8 (± 0.1)	5.8 (± 0.1)	Lys133 (NZ)	2.8 (± 0.1)	2.8 (± 0.1)	2.7 (± 0.1)
Asn57 (ND2)	2.9 (± 0.1)	7.6 (± 0.1)	6.7 (± 0.1)	Arg163 (NE)	2.7 (± 0.1)	3.2 (± 0.2)	2.8 (± 0.1)
				His197 (NE2)	2.7 (± 0.1)	2.7 (± 0.1)	2.7 (± 0.1)

**Table 2:** Binding free-energy (in kcal.mol^−1^) values obtained from MM/GBSA, MM/PBSA, and SIE approaches in kcal·mol^−1^.

	BPH1	BPH2	BPH3
Experimental	−8.82 ^a,b^	−7.0 ^a,c^	(n/d)
MM/GBSA	−96.07	−71.72	−82.96
MM/PBSA	−107.09	−82.64	−110.73
SIE	−13.53	−13.49	−14.37

^a^ Results converted to kcal.mol^−1^ based on inhibition constants obtained against KDO8P synthase from ^b^ *E. coli* [31] and ^c^ *N. meningitidis* [22]. (n/d) Not Determined.

## Supplementary Materials

The following are available online, Figure S1: Ramachandran Favoured (91.82%), Ramachandran Outliers (2.6%) Rotamers Outliers (4.41%), Figure S2: Non-local Atomic Interaction Energy obtained from Anolea and the protein model quality obtained from Qmean, Figure S3: A) Overall model quality and B) Local model quality obtained from ProSA-web, an interactive web service for the recognition of errors in three-dimensional structures of proteins https://prosa.services.came.sbg.ac.at/prosa.php, Figure S4: Overall quality factor evaluated by ERRAT, Figure S5: Re-docking of A) PEP and A5P substrates and B) KDO8P product. At the bottom are the RMSD (in Å) and docking energies (in kcal/mol), Figure S6: Overlapped structures of the bisphosphate inhibitors with PEP and A5P substrates (PDB ID: 1FWW). The carbon atoms of the substrates are colored purple. A, B and C are the superimposition with BPH1, BPH2 and BPH3, respectively.
