# Supplementary material for: Computational Investigation of Bisphosphate Inhibitors of 3-Deoxy-d-manno-octulosonate 8-phosphate Synthase
Source: Molecules. 2019 Jun 27;24(13):2370. doi: 10.3390/molecules24132370 (PMC6650799; doi:10.3390/molecules24132370)
Supplement: Supplementary file 1 [file molecules-24-02370-s001.pdf]

Computational Investigation of Bisphosphate Inhibitors of 3-Deoxy-D-manno-octulosonate 8-phosphate Synthase

Jéssica de Oliveira Araújo [1], Alberto Monteiro dos Santos [1], Jerônimo Lameira [1], Cláudio Nahum Alves [1], and Anderson Henrique Lima [1,*]

[1] Laboratório de Planejamento e Desenvolvimento de Fármacos, Universidade Federal do Pará, Belém, Brasil.
***** Correspondence: anderson@ufpa.br;

SUPPORT INFORMATION

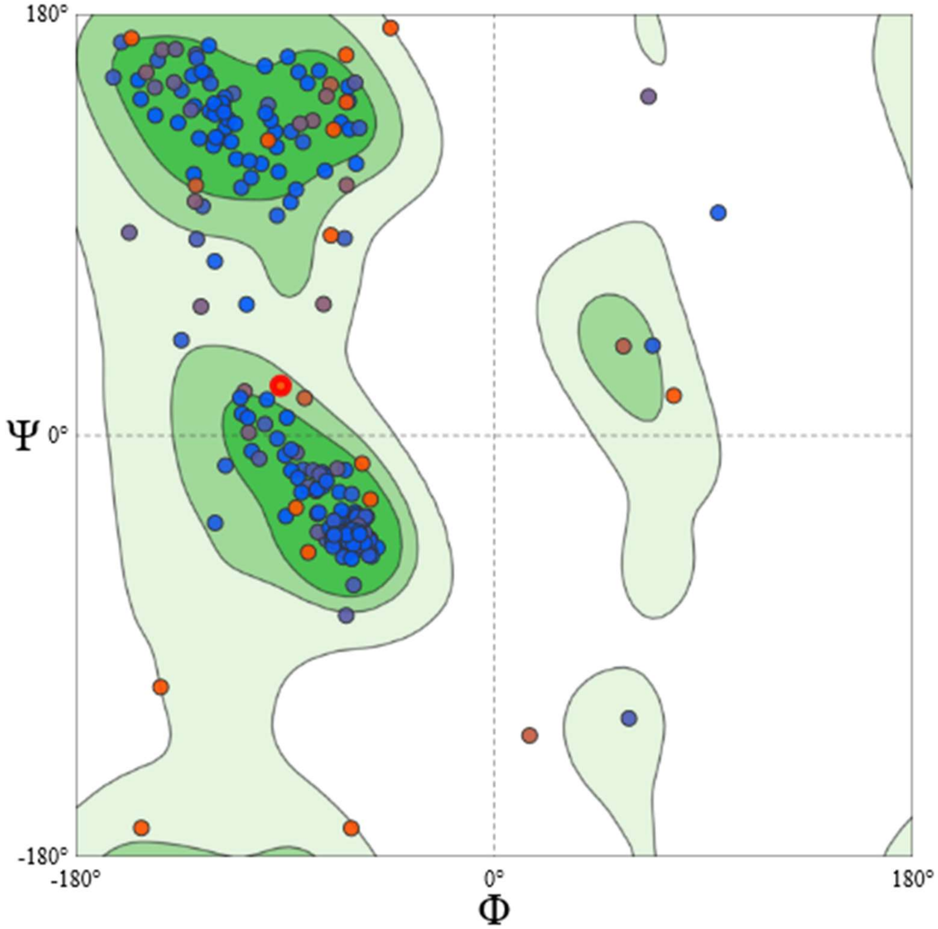

Figure S1. Ramachandran Favoured (91.82%), Ramachandran Outliers (2.6%) Rotamers Outliers (4.41%).

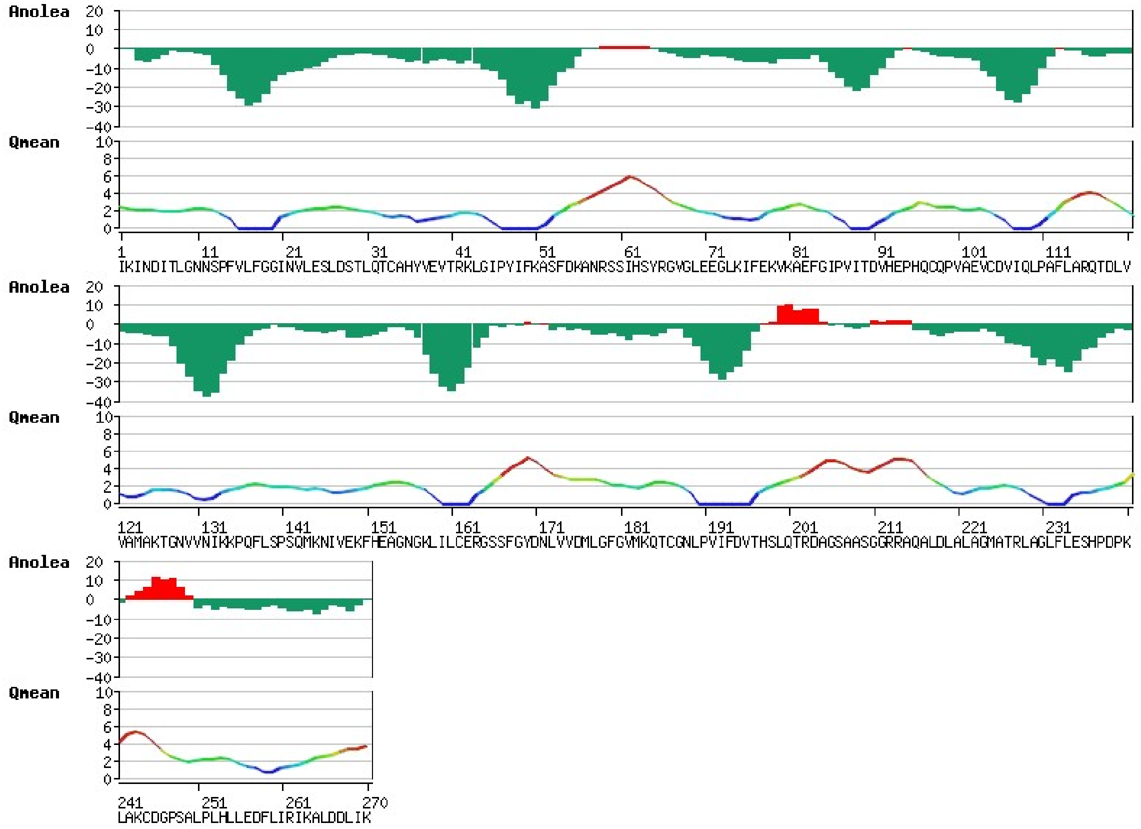

Figure S2. Non-local Atomic Interaction Energy obtained from Anolea and the protein model quality obtained from Qmean.

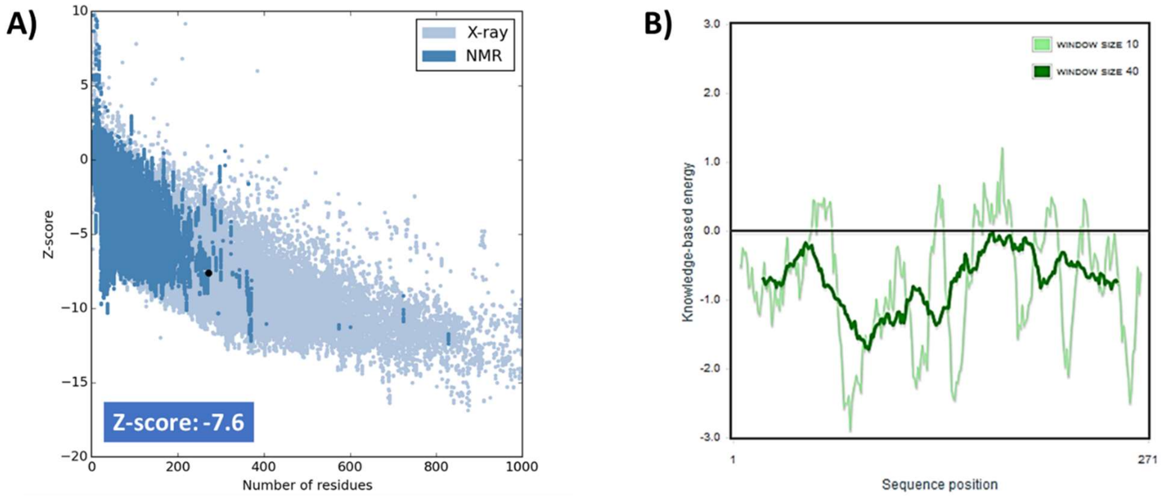

Figure S3. A) Overall model quality and B) Local model quality obtained from ProSA-web, an interactive web service for the recognition of errors in three-dimensional structures of proteins https://prosa.services.came.sbg.ac.at/prosa.php.

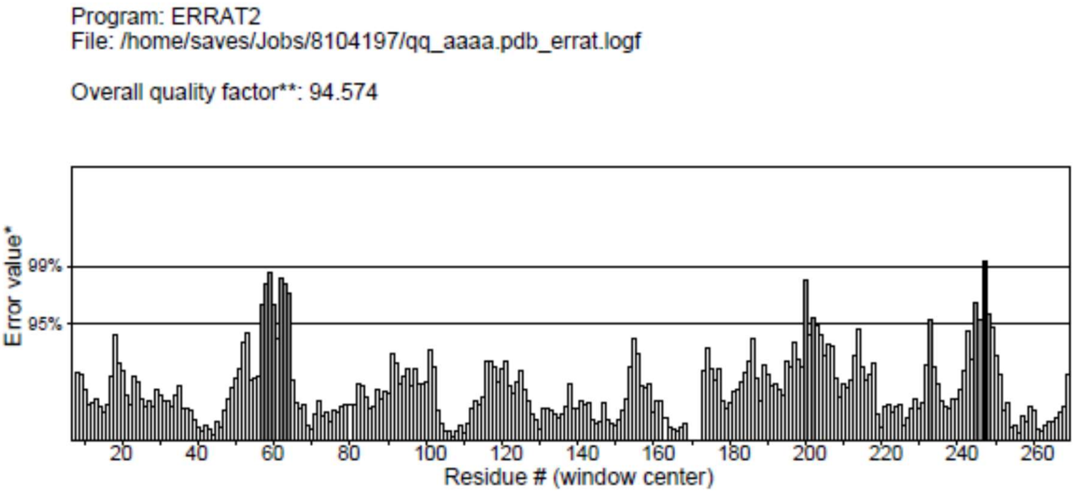

Figure S4. Overall quality factor evaluated by ERRAT.

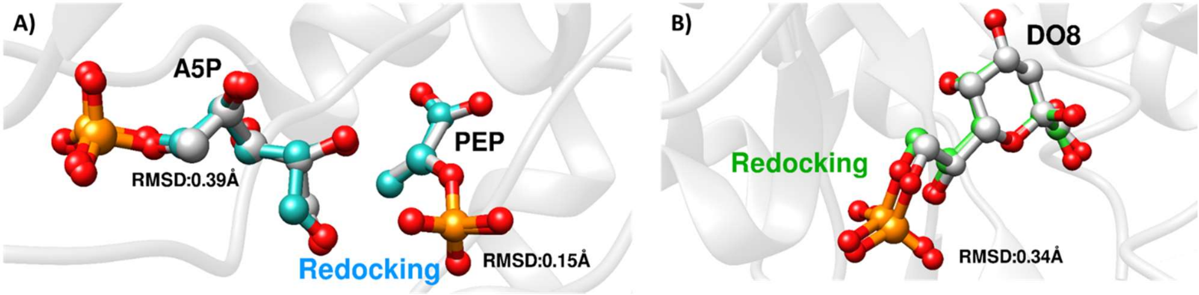

1FWW							
A5P				PEP			
RMSD	MolDock Score	Rerank Score	Hbond	RMSD	MolDock Score	Rerank Score	Hbond
0.394Å	-79.18	-63.51	-23.43	0.153	-111.61	-96.74	-21.96

1X6U			
Product KDO8P (DO8)			
RMSD	MolDock Score	Rerank Score	Hbond
0.345Å	-79.76	-69.39	-13.99

Figure S5. Re-docking of A) PEP and A5P substrates and B) KDO8P product. At the bottom are the RMSD (in Å) and docking energies (in kcal/mol).

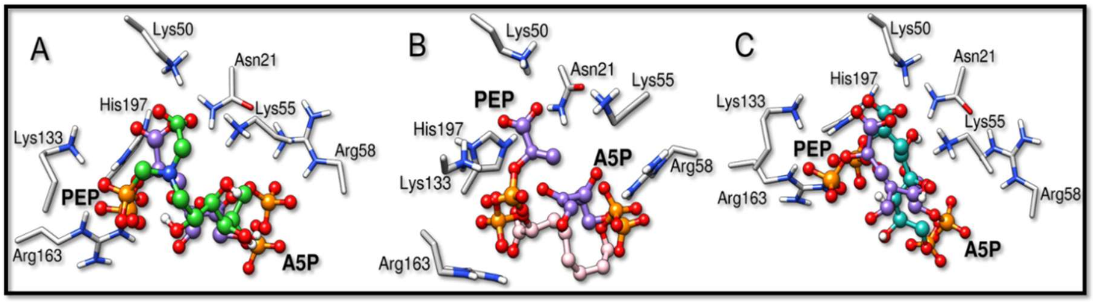

Figure S6. Overlapped structures of the bisphosphate inhibitors with PEP and A5P substrates (PDB ID: 1FWW). The carbon atoms of the substrates are colored purple. A, B and C are the superimposition with BPH1, BPH2 and BPH3, respectively.
